# Effect of 3D Printing Parameters on the Transparency of Medical Hydrogels for Corneal Stroma Fabrication

**DOI:** 10.3390/gels11070528

**Published:** 2025-07-08

**Authors:** Qiang Gao, Kaicheng Yu, Youyun Shang, Zexue Lin, Min Zhu, Lihua Lu, Tao Jiang, Peng Zhang

**Affiliations:** 1 School of Mechatronics Engineering, Harbin Institute of Technology, Harbin 150001, China; 2 Chongqing Research Institute of HIT, Chongqing 401121, China; 3 School of Electrical Engineering and Automation, Harbin Institute of Technology, Harbin 150001, China

**Keywords:** cornea stroma, transparency, 3D bioprinting, collagen, process parameter

## Abstract

Medical hydrogels represent a promising solution for the treatment of corneal diseases and trauma, offering potential to address the shortage of donor corneas. To meet the functional requirements of artificial corneas in tissue engineering, it is crucial to fabricate biomimetic structures with high optical transparency using 3D printing techniques. As fiber alignment during the printing process has a pronounced impact on light transmittance, precise control of the printing parameters is essential. This study focuses on the experimental optimization of 3D printing conditions for hydrogel materials to improve their physical properties, particularly optical clarity, thereby enhancing their suitability for artificial corneal applications. Collagen derived from bovine Achilles tendons was chosen due to its excellent printability. A series of controlled experiments were conducted to systematically investigate the influence of key process parameters on hydrogel transparency. The findings enabled the identification of an optimized parameter set that significantly improved the optical properties of the 3D-printed biomimetic corneal stroma. Additionally, cell seeding and culture assays confirmed the favorable biocompatibility of the developed material.

## 1. Introduction

As a transparent film located in the front of the eye, the cornea is an important optical element of the dioptric system of the eye to transmit and refract visible light [1]. Trauma, bacterial and viral infections, and heritable diseases of the cornea can cause irreversible decline or loss of its optical function [2,3], which consequently leads to permanent vision impairment or blindness [4,5]. However, due to the shortage of cornea donors, only 12,000 patients can find cornea donors annually, which results in millions of patients cannot be cured [6]. Additionally, the percentage of long-term failure of the corneal transplantation can exceed 30% [7]. Once rejection happens, successful acceptance of a new cornea may encounter a higher risk.

The rapid development of three-dimensional (3D) printing technology and tissue engineering has brought hope for fabricating in vitro functionalized artificial corneal tissue, which is a promising solution to alleviate this situation [8]. Among the various biomaterials explored for artificial corneal fabrication, medical hydrogels, especially collagen-based hydrogels, have emerged as core candidates due to their excellent optical transparency, biocompatibility, and structural tunability [9]. It can not only prevent immune rejection response but also fabricate a patient-specific artificial cornea with customized curvature and thickness by additive manufacturing process, which has merits of high versatility, repeatability, and reproducibility [10,11].

Numerous meaningful researches have been conducted to promote the in vitro fabrication of artificial corneal tissue [12,13]. Liu et al. [14] developed corneal stroma from cross-linked collagen, which has better optical clarity than that of native human cornea. The corneal stroma remained transparent after implanting into rabbit and porcine for six months. Wu et al. [15] printed the human corneal epithelial cells with hybrid ink of collagen, gelatin, and alginate. The printed cells demonstrated good cellular viability and great capacity to proliferate after printing. Anni et al. [16] validated the feasibility of 3D laser-assisted bioprinting for fabricating corneal structures utilizing human stem cells. The fabricated layered cornea-mimicking tissues have good mechanical properties without additional crosslinking. Abigail et al. [17] produced collagen-based corneal stroma equivalents based on the 3D topographic data of an adult human cornea. It exhibited excellent viability of 83% for corneal keratocytes at day 7 post-printing.

Despite the above progress in this field, manufacturing artificial corneas with comprehensive physiological functions [8] of the native human cornea, such as mechanical strength, optical transparency, morphology, and gene expression, remains extremely challenging. The research on cornea structure is the prerequisite to mimic its physiological functions. It has become evident that the cornea consists of three major layers: an epithelium, a stroma, and an endothelium, and the stroma is the primary element that accounts for approximately 90% of corneal thickness [1,6]. According to the research on the microscopic architecture of the stroma [18,19], it consists of many collagenous lamellae, which are parallel with the corneal surface, and keratocytes are populated sparsely in the stroma. The collagen fibrils are parallel arranged in individual lamellae, while the orientation of the filaments varies between the adjacent lamellae. The microscopic orientation and arrangement of collagen fibrils and lamellae determine the biomechanical and optical properties of stroma [20]. Therefore, emulating the microscopic arrangement of the collagen fibrils may be advantageous for fabricating artificial corneas with comprehensive physiological functions.

Many efforts have been implemented to mimic the microscopic structural characteristics of the natural human cornea by controlling the arrangement of collagen fibrils [21,22]. Jim et al. [23] found that the orientation of collagen fibrils can be aligned by a horizontal magnetic field, by which corneal stroma scaffold composed of orthogonally oriented collagen fibrils can be reconstructed. Kim et al. [24] utilized the shear stress during the extrusion of filaments to control the orientation of collagen fibrils, by which the impact of the nozzle diameter and flow rate on the transparency was investigated. Some novel methods to produce collagen materials also have been developed. Gervaise et al. [25] proposed a new method to produce large-scale dense hierarchical ordered collagen matrices from rat tail tendon, which has potential application to develop highly enriched collagen matrices for cornea stroma substitutes. Jian et al. [26] proposed a collagenous matrix with uniform fibrillar diameter and inter-space. The orientation feature of fibrils is similar to that of native stroma, which indicates the feasibility that the corneal tissue with spatially complex collagen-based structure could be produced under the bottom-up strategy. Hyeonji et al. [27] developed a new bioink of decellularized extracellular matrix from bovine cornea. It not only shows higher transparency than human cornea but also has excellent biocompatibility and printability for extrusion 3D printing, which has potential application to fabricate tissue-engineered cornea.

The above reviews indicate that the shear stress during the extrusion can orientate the collagen fibrils, which has a positive effect on improving the transmittance. Adjusting the process parameters of extrusion 3D printing may be an effective mean to improve the corneal transmittance. This study aims to fabricate corneal stroma with enhanced optical transmittance using a self-developed collagen-based hydrogel, highlighting the pivotal role of hydrogel material optimization in achieving functionally biomimetic corneal substitutes. The influence of printing process parameters on the light transmittance of the printed corneal stroma was investigated experimentally. This research is helpful in improving the optical performance of the printed corneal stroma.

## 2. Results and Discussion

### 2.1. The Influence of Process Parameters on the Transmittance

During the printing process, the temperature of the printing head was set at 4 °C to avoid the impact of the temperature on the material properties of collagen. Besides, the platform temperature of the printing plate was set at 0 °C to minimize the viscoelastic deformation of printed filaments. The printing parameters may affect the transparency of the printed corneal stroma, mainly including the angle between the adjacent layers (ABAL), the nozzle diameter, and the fusion rate of adjacent filaments (FRAF). The printing parameters to be investigated in the research are listed in Table 1.

#### 2.1.1. The Influence of the Collagen Concentration

**Figure 1:**
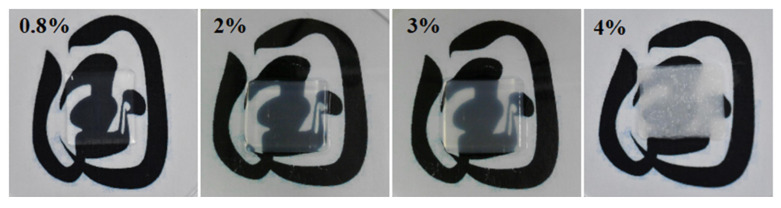
The printed corneal stroma with varying collagen concentrations.

Figure 1 presents the images of the printed corneal stromas with varying collagen concentrations of 0.8%, 2%, 3%, and 4%, respectively. It can be observed visually that the transparency of the printed corneal stroma declines with the increase of the collagen concentration when the other printing parameters are identical.

**Figure 2:**
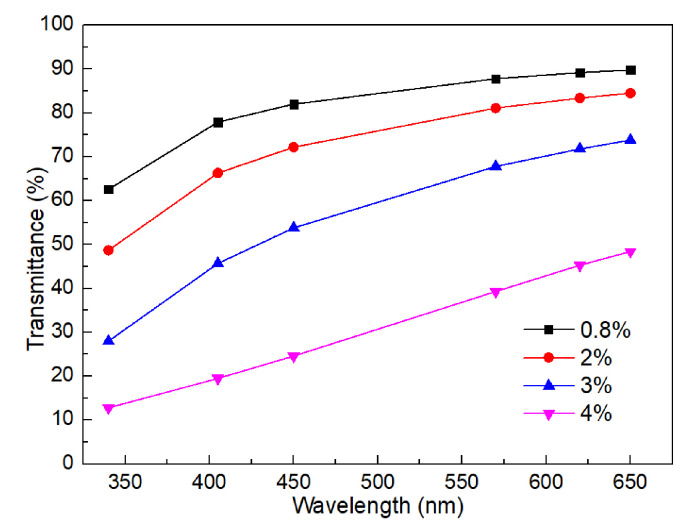
Light transmittance at different wavelengths of the visible light spectrum.

To evaluate their transparency quantitatively, their transmittance at different wavelengths of the visible light spectrum was further tested and compared, as demonstrated in Figure 2. It depicts that the transmittance drops from 89.8% to 48.4% when the collagen concentrations increase from 0.8% to 4%. Besides, the transmittance of the printed corneal stroma increases with the increase of the visible light wavelengths. It indicates that the developed collagen materials show poor light transmittance when their collagen concentration is higher than 3%, which is not suitable for printing the corneal stroma.

**Figure 3:**
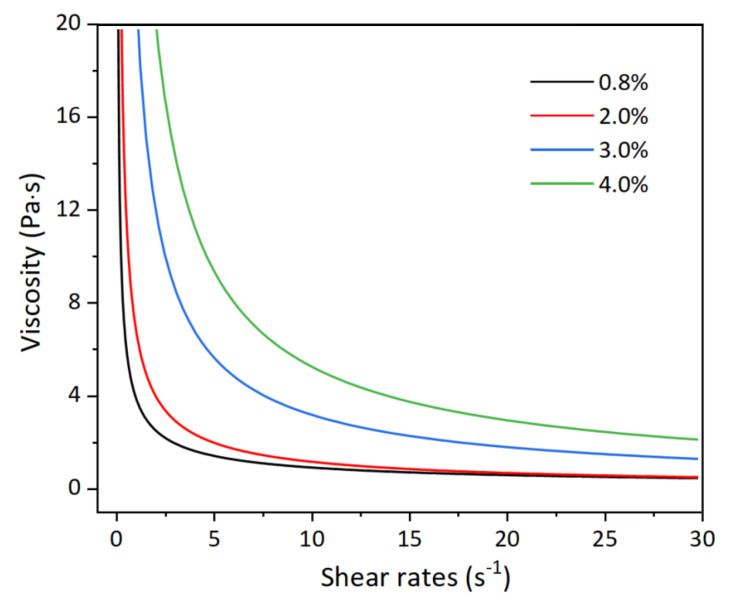
Viscosity curve of collagen with varying shear rate and concentration.

The above phenomenon is mainly attributed to the rheological properties of collagen materials. The obtained viscosity curves with the variation of shear rates are presented in Figure 3. A remarkable distinction among the collagen viscosity curves at various concentrations, with material with a lower concentration indicating a lower viscosity. This is because the lower concentration means that the collagen molecules have less chance to entangle with each other. Since the collagen ink is a polymer solution, the weakened entanglement would restrict the formation of polymer networks, which leads to a decreasing viscosity of collagen materials. The decreased viscosity enhances the fluidity of collagen ink, which leads to a smooth and flat surface of 3D printed architecture. This eventually results in a better light transmittance of corneal stroma.

#### 2.1.2. The Influence of the ABAL

**Figure 4:**
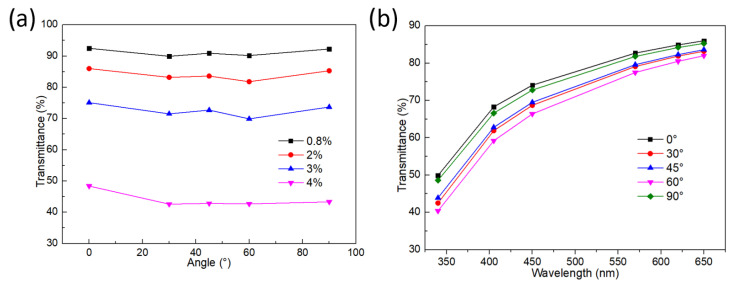
The influence of the ABAL on the transmittance. (**a**) Light transmittance of different concentration with varying ABAL, (**b**) Light transmittance of various ABAL with varying wavelength.

The ABAL affects the arrangement direction of collagen fibrils and the surface quality of the printing layer, which will further affect the transmittance. Figure 4a compares the transmittance of the printed corneal stroma with different ABAL from 0° to 90° for visible light wavelengths of 650 nm. The other printing parameters are identical to those listed in Table 1. The ABAL of 0° represents the orientation of the fibrils are parallel, while the ABAL of 90° represents the orientation of the fibrils are perpendicular. The ABAL has a similar influence on the transmittance of corneal stroma with different collagen concentrations. The corneal stroma shows higher transmittance when the ABAL is 0° or 90°. However, its transmittance decreases significantly when the ABAL is 30°, 45° or 60°, and it reaches a minimum when the ABAL is 60°. Furthermore, Figure 4b presents the transmittance of 2% collagen concentration for different visible light wavelengths.

The above phenomenon is mainly attributed to the fact that the collagen fibrils are gradually oriented along the flow direction under the shear stress in the extrusion process. The microscopic structure of the corneal stroma is arranged in a regular way when the ABAL is 0° or 90°. When the visible light incident, it can produce destructive interference in other directions except the incident direction and finally show a transparent state. However, the collagen fibrils inside the collagen stroma are disordered when the ABAL is set to other values. Consequently, the visible light incident cannot produce destructive interference in other directions except the incident direction, which results in excessive light intensity loss in the collagen stroma, and in turn decreased transparency.

Although the ABALs of 0° and 90° exhibit similar levels of transparency, the printed constructs with 90° ABAL demonstrate significantly higher mechanical strength and shape fidelity. This is because at 90°, the filaments in adjacent layers are deposited perpendicularly, forming a crosshatched structure that improves mechanical interlocking and supports better dimensional stability. In contrast, a 0° ABAL results in all filaments being aligned in the same direction, making the structure more prone to spreading and deformation due to filament swelling and reduced interlayer cohesion. Therefore, an ABAL of 90° is preferable for achieving structurally robust and geometrically accurate corneal stromal scaffolds.

#### 2.1.3. The Influence of the Nozzle Diameter

When investigating the influence of the nozzle diameter on the transmittance, the match between the printing thickness of each layer and the nozzle diameter should be considered especially. Because the suitable printing thickness of nozzles with different diameters is different, if the printing thickness is inappropriate, it will affect the filament formation and thus affect the mechanical properties and optical performance of collagen matrix.

**Figure 5:**
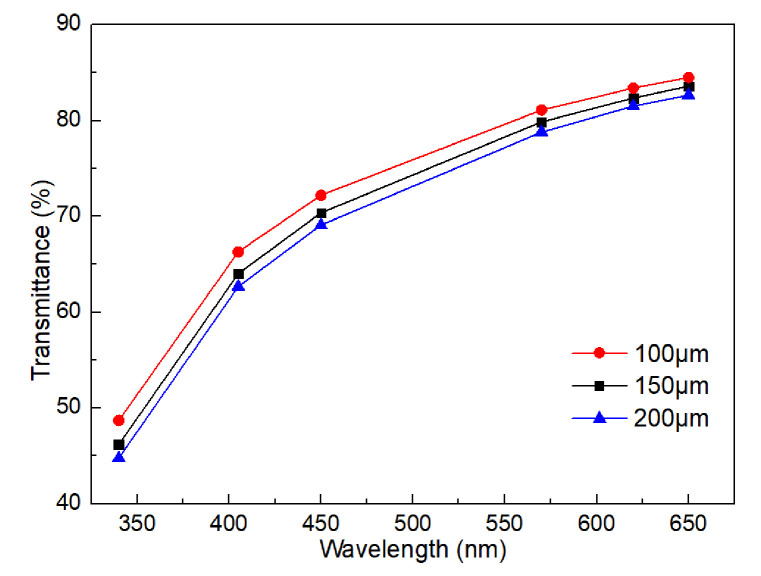
Light transmittance with different nozzle diameter.

Figure 5 presents the transmittance of prepared collagen concentration printed by different nozzle diameters of 100 μm, 150 μm, 200 μm. It indicates that the nozzle diameter of 100 μm shows best transmittance, while the nozzle diameter of 200 μm is worst. This phenomenon demonstrates that the smaller the nozzle diameter, the better the light transmittance of the printed collagen matrix. This is because the smaller the nozzle diameter is, the greater the shear force in the flow field, and the collagen molecules and filament tend to be regularly oriented along the flow direction under the action of shear force.

#### 2.1.4. The Influence of the FRAF

Since the cornea stroma is a nonporous 3D architecture, the filaments must be tightly fused during the 3D printing process. Specifically, during the deposition of filaments in the same layer, no gap is permitted between adjacent filaments. The structure consisted of well-arranged filaments, which would lead to enhanced transmittance. However, the slight grooves that occurred at the fusion of parallel filaments would result in an uncontrollable surface of cornea stroma. This might impact the transmittance of the entire 3D structure. Therefore, the FRAF is defined as the ratio of the volume of the fusion part to that of the original filament. By regulating the relative location of adjacent filaments, the value of FRAF can be set to various values.

**Figure 6:**
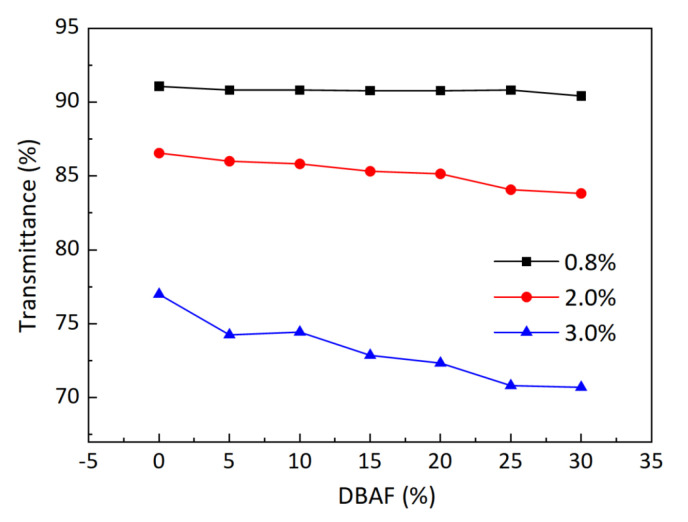
Light transmittance with various FRAF.

Figure 6 presents the transmittance of 0.8%, 2%, and 3% collagen concentration printed by FRAF from 0% to 30% with an interval of 5%. The printing trials were conducted with the ABAL of 90° and nozzle diameter of 100 μm. It can be seen that the FRAF has little influence on the transmittance when the collagen concentration is 0.8%. While the FRAF curves for the collagen concentration of 2% and 3% demonstrate remarkable shrinkage with the increasing FRAF. When the adjacent filaments fused with each other, the local thickness at the fusion would increase during the 3D printing process. This directly leads to a shrinkage of transmittance. According to the above experimental results, the FRAF during the fabrication of 3D architectures should be controlled to a low value.

Generally, the collagen concentration, the ABAL, the nozzle diameter, and the FRAF of the process parameters have a relatively large effect on the light transmittance of the collagen matrix. When the ABAL is 90°, the optical and mechanical performance of time mechanics is the best. The influence of collagen concentration and FRAF on the transmittance is unidirectional, and the greater the value, the smaller the transmittance. The optimized printing parameters for cornea stroma are eventually obtained in Table 2.

### 2.2. Mechanical Testing of 3D Printed Cornea Stroma

To evaluate the mechanical performance of the collagen scaffolds, uniaxial tensile tests were conducted on hydrogels with collagen concentrations of 0.8%, 2%, 3%, and 4%. As shown in Table 3, the Young’s modulus increased significantly with collagen concentration, rising from 26.9 Pa at 0.8% to 243 Pa at 4%. This trend reflects the enhancement of molecular entanglement and fibrillar density within the collagen network, which contributes to increased structural stiffness.

However, this mechanical improvement comes at the expense of optical performance. Among all tested formulations, only the 0.8% collagen scaffold achieved near-ideal transparency, with a transmittance of 89.8% at 700 nm. This reveals an intrinsic trade-off between optical clarity and mechanical robustness, primarily dictated by the internal microstructure of collagen hydrogels. At low concentrations (e.g., 0.8%), the loose fibrillar network reduces refractive index heterogeneity and minimizes light scattering, resulting in higher transparency. Nevertheless, the reduced entanglement and weaker inter-fibrillar interactions limit the scaffold’s load-bearing capacity.

In contrast, higher collagen concentrations (e.g., 3–4%) lead to a denser and more crosslinked fibrillar structure, which significantly improves mechanical stability and resistance to deformation—critical features for surgical handling. However, such dense matrices also introduce optical discontinuities, such as fibril clustering and anisotropic orientation, thereby increasing light scattering and reducing transparency.

Given this inherent antagonism, a strategic trade-off must be made. Although the 0.8% formulation does not meet the generally accepted tensile strength threshold (∼0.25 MPa) for suturable corneal grafts [2,3], it demonstrates excellent printability and geometric fidelity, and provides an ideal platform for structure–0function investigations with a focus on transparency. Therefore, 0.8% collagen was selected in this study to prioritize optical function, which is central to corneal physiology. In the future, the development of novel biomaterials is expected to enable post-printing enhancements—through physical (e.g., UV or thermal treatment), chemical crosslinking, or the integration of multilayered or composite architectures—that simultaneously improve both mechanical strength and optical transparency, thereby facilitating clinical translation.

### 2.3. Biocompatibility of 3D Printed Cornea Stroma

**Figure 7:**
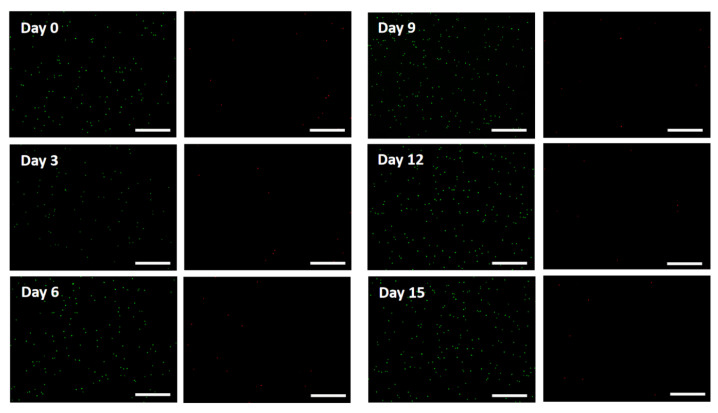
Fluorescence microscopy of the L-929-seeded scaffolds. Scale bar: 100 μm.

To verify the biocompatibility of the fabricated cornea stroma, L-929 cells with the cellular density of $2\times 10^{6}$ cells/mL were separately added into the prepared 0.8% collagen solution and 3D printed with the optimized 3D printing parameters. Each cell-seeded cornea stroma was cultured with MEM and treated inside a cell culture incubator, with 5% CO_2_ and 95% relative humidity, for 12 days. The Live/Dead assay results are demonstrated in Figure 7.

**Figure 8:**
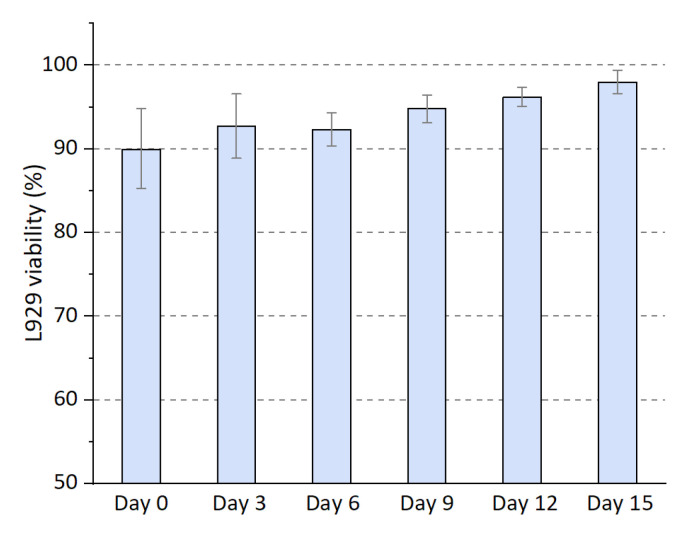
Cell viability (%) of L-929 cultured within the bioprinted scaffolds. Results are based on Live/Dead assay.

To further investigate the biocompatibility of the presented cornea stroma, the cellular viability of L-929 has been quantified based on the analysis with the results demonstrated in Figure 8. For each kind of cell, the cellular viability indicates a lower value of around 90% at day 0, revealing that the shear stress generated during the extruding process in the nozzle induces cell death. The faster flow near the wall of the printing nozzle usually leads to a higher shear stress, which results in cell deformation and ultimate death [28]. As the culturing process of each cell-seeded scaffold, the cellular viability shows a remarkable increase, thus suggesting that the diffusion of nutrients is sufficient to guarantee cellular growth. This demonstrates the splendid biocompatibility of the 3D printed cornea stroma with the optimized 3D printing parameters.

While the L-929 fibroblast cell line used in this study is not specific to corneal tissue, it was selected as a standard model for preliminary biocompatibility evaluation due to its widespread adoption, robust growth characteristics, and well-documented sensitivity to cytotoxic stimuli. This choice allowed us to assess baseline cellular compatibility of the printed hydrogels without introducing additional biological complexity associated with primary stromal or stem cell culture. We acknowledge, however, that L-929 cells cannot fully replicate the biological behavior of corneal stromal keratocytes or limbal stem cells. Therefore, the findings of this study primarily reflect general cytocompatibility rather than tissue-specific cellular responses. Future work will incorporate more physiologically relevant corneal cell types to further validate the suitability of the scaffolds for clinical corneal reconstruction applications.

### 2.4. Discussion

To validate the effectiveness of the proposed strategy, we compared the findings of this study with recent advances in corneal stroma bioprinting. Although prior research has demonstrated the feasibility of constructing transparent corneal scaffolds using a variety of bioinks—such as methacrylated gelatin (GelMA), decellularized extracellular matrix (dECM), and collagen–agarose composites—these materials often require chemical crosslinkers, complex formulations, or intricate post-processing steps [29]. In contrast, the collagen bioink used in this study was derived from bovine Achilles tendons through a simplified and reproducible extraction protocol, without any chemical additives or crosslinking agents. This approach preserves the natural shear-induced alignment behavior of collagen fibers and provides a cleaner material basis for evaluating structural and optical performance.

Moreover, we systematically and quantitatively assessed four key printing parameters—collagen concentration, ABAL, nozzle diameter, and (FRAF—which are mentioned in previous studies but rarely investigated in a unified framework. For example, Kim et al. [24] studied the shear-aligned fibrils within dECM hydrogels and their impact on transparency, but did not independently analyze the effects of ABAL or filament fusion. Similarly, Kutlehria et al. [30] evaluated the transmittance of different hydrogel compositions, yet did not explicitly control inter-filament merging or nozzle-induced shear stress. Under the optimized configuration (0.8% collagen, 90° ABAL, 100 μm nozzle, 0% FRAF), our constructs achieved a transmittance of 89.8% at 700 nm, which is comparable to or even higher than that reported for GelMA- or dECM-based structures (typically 75–85%). In addition, this study introduces and quantitatively defines FRAF as a novel parameter that strongly correlates with local thickness variations and optical loss—an aspect that has not been previously addressed in the literature.

Furthermore, the use of chemically unmodified collagen and shear-guided filament orientation provides a clinically relevant strategy for fabricating fully biocompatible, optically clear scaffolds that can be potentially adapted for corneal transplantation without the need for toxic reagents or extensive post-processing.

Finally, the optimized constructs also exhibited excellent biocompatibility, maintaining over 90% cell viability of L-929 fibroblasts after 12 days of culture. This result further confirms that shear-induced fiber alignment, combined with parameter tuning, enables the synergistic optimization of mechanical strength, optical clarity, and cellular performance within a single-step bioprinting process. Taken together, the study not only provides an optimized protocol for transparent corneal scaffold fabrication but also proposes a unified framework for correlating process mechanics with tissue-level optical function—advancing both the methodology and its biomedical applicability.

## 3. Conclusions

In this research, a collagen-based hydrogel scaffold was utilized as the core material for fabricating 3D printed corneal stroma, and the influence of printing process parameters on its optical performance was systematically investigated. Printing trials and transmittance tests were conducted to determine the optimal parameters that enhance the transparency of the collagen constructs. In our research, a novel method was developed for extracting collagen materials from bovine Achilles tendon, enabling the preparation of medical-grade hydrogels with improved 3D printability suitable for biomimetic corneal scaffolds. Then, 3D printing trials demonstrated that the optical transparency of the collagen-based stroma can be significantly enhanced by regulating key process parameters, including nozzle diameter, ABAL, and FRAF. Meanwhile, an optimized parameter set—nozzle diameter of 100 μm, ABAL of 90°, and FRAF of 0%—was identified for achieving the highest transparency using 0.8% collagen hydrogel. At last, L-929 cells were successfully cultured on the 3D printed transparent collagen scaffolds, and their high viability confirmed the excellent biocompatibility of the collagen-based hydrogel material.

## 4. Materials and Methods

### 4.1. Collagen Material Preparation

Collagen was selected as the base material in this study due to its prominence as the primary structural component of the native corneal stroma, its excellent shear-induced alignability, and its proven printability. Compared to hyaluronic acid, which lacks sufficient mechanical strength and shape retention during extrusion, collagen offers better filament fidelity and structural integrity for 3D bioprinted corneal constructs. The preparation process of collagen material is tedious and complicated, and the dimension uniformity of the collagen fibers is difficult to control. The commercialized collagen materials are generally not only expensive but also show poor printability. To overcome the limitation of commercialized collagen materials, a novel method for preparing collagen materials from bovine Achilles tendon is proposed in this paper. The prepared collagen materials have excellent printability for extrusion printing. The specific preparation process is as follows:

#### 4.1.1. Pretreatment of Fresh Bovine Achilles Tendon

The fresh bovine Achilles tendons were first washed to remove the fascia and grease and then frozen at −20 °C. Then, the tendons were cut into thin slices and mashed in a tissue masher. Following sequentially by washing with distilled water at normal temperature, soaking in 0.8% sodium carbonate solution for 12 h, and soaking in 0.8% sodium carbonate at 4 °C for 12 h, rinsing with distilled water, fully dried and frozen at −20 °C, the raw materials for collagen preparation were acquired.

#### 4.1.2. Crude Extraction of Collagen

Soaking the raw materials acquired in the previous step into 1.2% acetic acid solution containing 2.7 mg/mL pepsin, then stirring evenly in a 4 °C water bath for extraction for more than 48 h, followed by centrifugation at 4 °C for 40 min with the speed set to 10,000 r/min, preserving the supernatant and discarding the precipitation.

#### 4.1.3. Salting Out

Mixing the supernatant with an equal volume NaCl solution with a concentration of 0.2 g/ mL, then standing for 12 h, followed by centrifugation for 40 min with the speed set to 10,000 r/min, preserving the precipitation and discarding the supernatant.

#### 4.1.4. Fine Extraction of Collagen

Soaking the precipitate obtained in the previous step into 1.2% acetic acid solution and stirring evenly in the 4 °C water bath for 48 h, centrifuging at 10,000 r/min for 40 min, preserving the supernatant, and discarding the precipitation. Repeat the steps in Section 4.1.3 as well as Section 4.1.4 to remove the miscellaneous proteins in collagen.

#### 4.1.5. Dialysis

Collecting the flocs obtained after the last salting out into a dialysis bag and placing in 1% acetic acid solution, changing the acetic acid solution every 24 h until the collagen in the dialysis bag expanded evenly, and no precipitation could be observed by dropping the silver nitrate solution into it.

#### 4.1.6. Dehydrate and Regulate the PH of Collagen

Dehydrating the dialysis bag in polyethylene glycol and then soaking it in 4 °C distilled water, changing the distilled water every 24 h until the PH of collagen reaches approximately 4.5. After the above steps were accomplished, the collagen in the dialysis bag was stored in an airtight container for constant temperature preservation at 4 °C.

To enhance the shape stability and mechanical strength of the printed corneal stroma, we further modulated the pH of the constructs after printing. Specifically, the freshly printed scaffolds were immersed in 1× PBS, which gradually increased the environmental pH and promoted the reassembly of collagen monomers into a physically crosslinked fibrillar network. This process relies on electrostatic and hydrophobic interactions, which are sensitive to pH and temperature, respectively. Such pH-mediated fibril formation enabled us to reinforce the printed structures without the need for chemical crosslinkers, preserving both the bioactivity and optical clarity of the collagen matrix.

### 4.2. Preparation of Bioinks

To investigate the influence of collagen concentration on the optical properties of printed corneal stroma, bioinks with varying concentrations of type I collagen were prepared. Due to the unique rheological characteristics of high-molecular-weight polymeric materials such as collagen, an excessively high concentration can lead to overly high viscosity, resulting in nozzle clogging during extrusion. Conversely, if the concentration is too low, the viscosity may be insufficient to maintain filament integrity, leading to poor shape retention after deposition. Based on preliminary printing trials, we selected the concentration range that allowed for stable extrusion and continuous filament formation as the basis for our experimental investigation. Specifically, collagen solutions were formulated at four different concentrations: 0.8%, 2%, 3%, and 4% (*w*/*v*), using the extracted collagen material described in Section 4.1. Each solution was prepared by dissolving the purified collagen in 1% (*v*/*v*) acetic acid and gently stirred at 4 °C to ensure uniform dispersion. No additional crosslinkers or rheological additives were introduced to preserve the intrinsic properties of the collagen for experimental control.

For biocompatibility assays, a cell-laden bioink was prepared by suspending L-929 fibroblast cells into the 0.8% collagen solution at a density of $2\times 10^{6}$ cells/mL. The mixture was gently stirred in an ice bath to maintain cell viability and homogenously distribute the cells within the collagen matrix. The resulting bioink was immediately loaded into the cartridge for extrusion-based bioprinting using the optimized printing parameters.

### 4.3. Rheological Investigation

Collagen is a pseudoplastic fluid that shows complex rheological properties, of which the viscosity usually changes with various concentrations. The varying viscosity of collagen ink will result in impacts on fluidity, which ultimately leads to a change in transparency of the printed corneal stroma.

**Figure 9:**
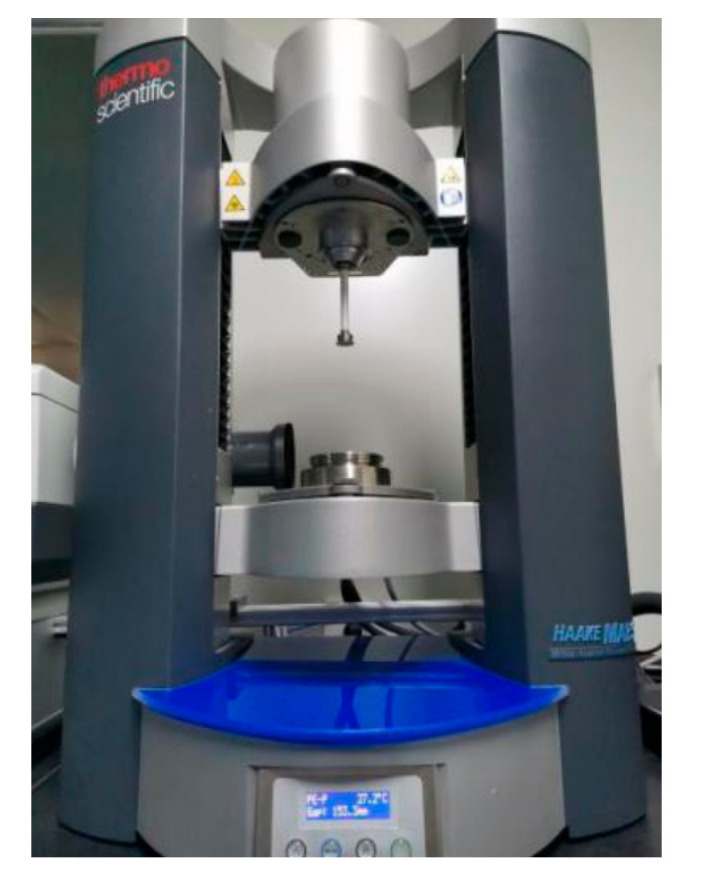
The rotation rheometer.

As demonstrated in Figure 9, a rotation rheometer (HAAKE MARS III, Thermo Fisher Scientific, Shanghai, China) was adopted for investigating the rheological properties of the proposed collagen material. The steady shear sweep analysis mode was performed from 1 to 30 s^−1^ to acquire the viscosity curves with various shear rates.

### 4.4. Mechanical Test

To evaluate the mechanical suitability of the proposed collagen for corneal applications, we conducted uniaxial tensile tests using a custom-assembled testing system comprising a high-precision pressure sensor (9256C1 SN1841599, KISTLER, Winterthur, Switzerland) and a linear translation stage (LX80-C 311A6, SELN, Dongguan, China). This setup enabled controlled uniaxial stretching of hydrogel specimens at a constant rate while accurately recording the force-displacement data. The Young’s modulus of each construct was determined from the linear region of the stress-strain curves. These mechanical tests were conducted to verify the structural integrity of the printed scaffolds and support their clinical relevance.

### 4.5. Transmittance Test

**Figure 10:**
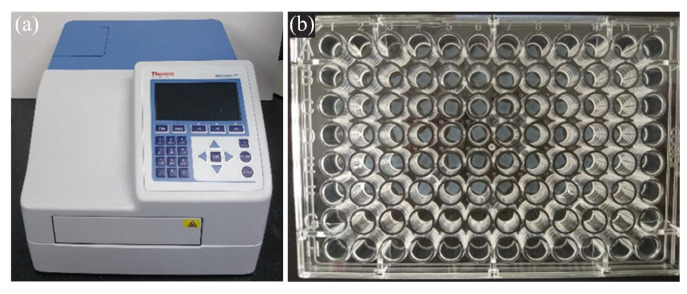
Experimental setup of transmittance test. (**a**) The microplate reader, (**b**) The well plate.

A microplate reader is employed in this research to quantitatively evaluate the transparency of the printed cornea stroma, as shown Figure 10. Its measurement principle is similar to that of the photoelectric colorimeter. When measuring, the tested samples are placed into the well plate, and the light waves from the light source will be filtered into monochromatic light. The photoelectric detector will convert the received optical signal into an electrical signal, which then will be amplified and sent to the microprocessor. Finally, the absorbance of the tested sample to the light of a certain wavelength can be calculated by Equation (1).

(1) $$T=\frac{I}{I_{0}}=\frac{1}{10^{A}}$$

where *A* represents the absorbance; $I_{0}$ and *I* denote the intensities of the incident and the transmitted monochromatic light, respectively; *T* is the transmittance of the tested sample.

### 4.6. Extrusion 3D Bioprinting Device

**Figure 11:**
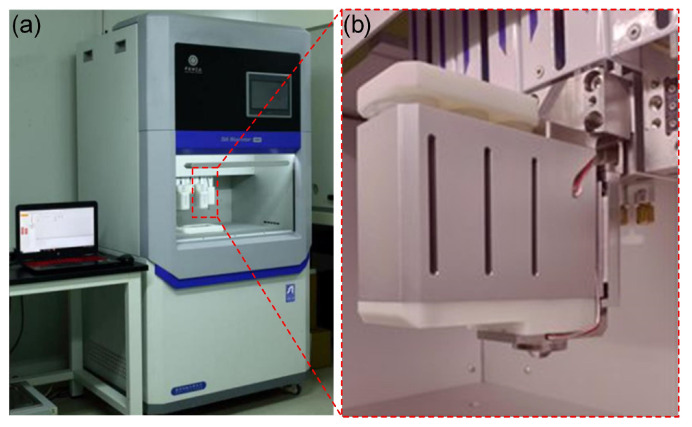
Experimental setup of printing trails. (**a**) A self-designed 3D bioprinter, (**b**) The extrusion-based 3D print head.

Figure 11 shows a homemade extrusion 3D bioprinter. It consists of three linear motion axis with position accuracy of ±5μm. It shows excellent temperature control capability with temperature control accuracy of ±0.1 °C in the range of −4–40 °C, and the temperature of the cartridge, the nozzle, and the printing stage can be regulated separately. The optional nozzle sizes are in the range of 50–400 μm. The maximum pressure of the air supply source is 0.6 MPa.

### 4.7. Culture of Cell-Laden Constructs

To meet the strict requirement for future medical applications, the 3D printed cornea stroma should be armed with splendid biocompatibility. Since the histological structure of natural cornea stroma mainly consists of corneal keratocytes (corneal fibroblasts), a mouse fibroblast cell line (L-929) was employed in our research. These cells were cultured in minimum essential medium (MEM) (PM150410, Pricella) with 1% (*v*/*v*) of penicillin/streptomycin and 10% (*v*/*v*) Fetal Bovine Serum (FBS) (164210-50, Pricella, Wuhan, China). Culture conditions were controlled by a cell culture incubator set at 37 °C, with 5% CO_2_ and 95% relative humidity.

To confirm the viability of cells seeded on the surface of each cornea stroma, a Live/Dead^TM^ Viability/Cytotoxicity kit, based on calcein-AM (green, live cells) and ethidium homodimer-1 (red, dead cells), was adopted. A fluorescence microscope (AZ100, Nikon, Tokyo, Japan) was utilized for imaging the cell-seeded samples, and the ImageJ software, Version 1.54p (Fiji) was employed to calculate the cell viability.

### 4.8. Statistics

Data were analyzed utilizing single-factor analysis to verify the robustness of the experimental data from the mechanical tests. All statistical analyses were performed using Microsoft Excel (Seattle, WA, USA). The experimental results were considered statistically significant at a significance level of 0.05 (*p* < 0.05).

## Figures and Tables

**Table 1:** The collagen concentration and printing parameters for corneal stroma.

Parameter	Value
Collagen concentration	0.8%, 2%, 3%, 4%
ABAL	0°, 30°, 45°, 60°, 90°
Nozzle diameter	100μm, 150μm, 200μm
FRAF	0%, 5%, 10%, 15%, 20%, 25%, 30%

**Table 2:** The optimized 3D printing parameters for cornea stroma.

Parameter	Value
Collagen concentration	0.8%
ABAL	90°
Nozzle diameter	100μm
FRAF	0%

**Table 3:** Young’s modulus of collagen hydrogels at different concentrations.

Collagen Concentration	Young’s Modulus (Pa)
0.8%	26.9
2%	51.5
3%	87.1
4%	243

## Data Availability

The original contributions presented in this study are included in the article. Further inquiries can be directed to the corresponding author.
